# Performance of N-terminal-pro-B-type natriuretic peptide in critically ill patients: a prospective observational cohort study

**DOI:** 10.1186/cc7110

**Published:** 2008-11-06

**Authors:** Isaline Coquet, Michael Darmon, Jean-Marc Doise, Michel Degrès, Bernard Blettery, Benoît Schlemmer, Philippe Gambert, Jean-Pierre Quenot

**Affiliations:** 1 Medical Intensive Care Unit, Saint-Louis University Hospital, AP-HP, 1 Avenue Claude Vellefaux, Paris, 75010, France; 2 Medical Intensive Care Unit, Dijon University Hospital, 1 boulevard Jeanne d'Arc, Dijon, 21079 Dijon cedex, France; 3 Biochemistry Laboratory, Dijon University Hospital, 1 boulevard Jeanne d'Arc, Dijon, 21079 Dijon cedex, France; 4 Université Paris-7 Paris-Diderot, UFR de Médecine, 75010 Paris, France

## Abstract

**Introduction:**

The purpose of this study was to assess the accuracy of N-terminal-pro-B-type natriuretic peptide (NT-proBNP) as a diagnostic tool to recognize acute respiratory failure of cardiac origin in an unselected cohort of critically ill patients.

**Methods:**

We conducted a prospective observational study of medical ICU patients. NT-proBNP was measured at ICU admission, and diagnosis of cardiac dysfunction relied on the patient's clinical presentation and echocardiography.

**Results:**

Of the 198 patients included in this study, 102 (51.5%) had evidence of cardiac dysfunction. Median NT-proBNP concentrations were 5,720 ng/L (1,430 to 15,698) and 854 ng/L (190 to 3,560) in patients with and without cardiac dysfunction, respectively (*P *< 0.0001). In addition, NT-proBNP concentrations were correlated with age (ρ = 0.43, *P *< 0.0001) and inversely correlated with creatinine clearance (ρ = -0.58, *P *< 0.0001). When evaluating the performance of NT-proBNP concentrations to detect cardiac dysfunction, the area under the receiver operating characteristic (ROC) curve was 0.76 (95% confidence interval (CI) 0.69 to 0.83). In addition, a stepwise logistic regression model revealed that NT-proBNP (odds ratio (OR) = 1.01 per 100 ng/L, 95% CI 1.002 to 1.02), electrocardiogram modifications (OR = 11.03, 95% CI 5.19 to 23.41), and severity assessed by organ system failure score (OR = 1.63 per point, 95% CI 1.17 to 2.41) adequately predicted cardiac dysfunction. The area under the ROC curve of this model was 0.83 (95% CI 0.77 to 0.90).

**Conclusions:**

NT-proBNP measured at ICU admission might represent a useful marker to exclude cardiac dysfunction in critically ill patients.

## Introduction

B-type (brain) natriuretic peptide (BNP) is a neurohormone that is secreted in response to volume expansion and pressure overload of cardiac ventricles. It has been reported that measurements of BNP and N-terminal-pro-B-type natriuretic peptide (NT-proBNP) are sensitive and specific tests for the diagnosis of heart failure in the urgent care setting [1-6]. In addition, BNP measurement was shown to be superior to clinical judgment in the identification of cardiac heart failure, regardless of the threshold value [7]. However, BNP concentrations frequently are elevated in critically ill patients, and several pre-existing conditions, including advanced age and renal failure, might influence BNP concentration [4]. Few studies have addressed the diagnostic value of natriuretic peptides in the ICU. This preliminary study aimed to evaluate the accuracy of NT-proBNP measured at admission to the ICU as a marker of cardiac dysfunction in a heterogeneous group of critically ill patients. The secondary objective of this study was to evaluate the impact of age and creatinine clearance on the performance of NT-proBNP as a diagnostic marker.

## Materials and methods

### Patients

We prospectively included consecutive adult patients (> 18 years old) who were admitted to the medical ICU of the Dijon Hospital between 1 September 2003 and 31 March 2004. Patients with end-stage chronic renal failure (that is, patients with chronic renal failure requiring renal replacement therapy) were excluded from the study. This study was approved by our institutional review board (IRB), and each of the included patients or next of kin received written information and gave oral consent. Our IRB waived the need for written consent.

### NT-proBNP measurement

Plasma levels of NT-proBNP were measured with the Elecsys Electro-chemo luminescent assay (Elecsys 2010; Roche Diagnostics, Basel, Switzerland) according to National Committee for Clinical Laboratory Standards guidelines and International Federation of Clinical Chemistry and Laboratory Medicine quality specifications for BNP methods. This test had a coefficient of variations of 3.2% to 2.4%, from 175 to 4,962 ng/L, with an analytical range of 5 to 35,000 ng/L.

### Diagnoses

Cardiac dysfunction was defined as any degree of respiratory insufficiency (acute or acute on chronic hypoxemia requiring oxygen therapy or mechanical ventilation) caused by a systolic or diastolic cardiac failure that requires inotropic drugs and/or diuretics. Diagnoses were based on clinical and radiographic findings and were validated by an echocardiography performed in each of the patients. These diagnoses were validated by two of the authors (IC and J-PQ) at the time of patient discharge. These physicians were unaware of the NT-proBNP measurement results. Echocardiography was performed in each of the patients. Left ventricular (LV) volumes were measured in the apical four-chamber view with the area-length method. The LV ejection fraction (EF) was calculated with standard formulas. Systolic LV dysfunction was defined as an EF of less than 50%. In patients with conserved LV EF, the evidence for diastolic dysfunction was considered as a documented cardiac dysfunction. To evaluate diastolic function, the thickness of the septal and posterior walls of the left ventricle and its internal dimensions were measured. The transmitral flow velocity was measured with the use of pulsed-wave Doppler, and the peak velocities of the E wave and A wave were measured. The ratio of these velocities, the E-wave deceleration time, and the isovolumetric relaxation time were measured in order to detect diastolic dysfunction. Electrocardiogram (ECG) modifications were defined as ST changes or T-wave inversion. Acute renal failure was defined by creatinine clearance, which was calculated at ICU admission with the Cockroft-Gault formula. Simplified Acute Physiology Score II (SAPS II) and organ system failure (OSF) score were calculated at admission [8,9]. Sepsis was diagnosed by using the criteria developed at the American College of Chest Physicians/Society of Critical Care Medicine consensus conferences [10]. Individual organ failure was defined according to the OSF score [8].

### Statistical analysis

Results were reported as medians and quartiles (interquartile ranges) or as numbers (percentages). Categorical variables were compared by using the chi-square test or Fisher exact test, and continuous variables were compared by using the non-parametric Wilcoxon test or the Mann-Whitney test. Logistic regression analyses were performed to identify variables that were associated significantly with cardiac dysfunction, as measured by the estimated odds ratio (OR) with 95% confidence interval (CI). Variables yielding *P *values of less than 0.20 in the bivariate analyses were entered into a forward stepwise logistic regression model in which cardiac dysfunction was the variable of interest. The covariates were entered into the model with critical removal *P *values of 0.1. Colinearity and interactions were tested. The Hosmer-Lemeshow test was used to check goodness-of-fit of the logistic regression. A receiver operating characteristic (ROC) curve that depicted the relationship between the proportion of true positives and the proportion of false positives was drawn, depending on the prediction rule used to classify the patients as having cardiac dysfunction. A 2 × 2 table was calculated to determine the sensitivity and specificity. Cutoff values, which were defined as the threshold values that maximized the sum of sensitivity and specificity, were determined for each score with the ROC curves. The positive likelihood ratio (LH) was computed to further evaluate the test performance. Indeed, in order to depict the input of NT-proBNP more precisely, we computed the LH in order to describe its ability to modify probabilities of having a specific diagnosis (prevalence of the disease in a specific population, for example) before the result of a test is known (named pre-test probability) into a post-test probability, which takes the result of the test into account [11,12]. All tests were two-sided, and *P *values of less than 0.05 were considered statistically significant. Statistical tests were performed with the SAS 6.12 software package (SAS Institute Inc., Cary, NC, USA).

## Results

Between September 2003 and March 2004, 198 consecutive patients fulfilled the inclusion criteria. Patient characteristics are summarized in Table 1. Median NT-proBNP concentrations were 5,720 ng/L (1,430 to 15,698) and 854 ng/L (190 to 3,560) in patients with and without cardiac dysfunction, respectively (*P *< 0.0001). In addition, NT-proBNP concentrations were correlated with age (ρ = 0.43, *P *< 0.0001) and inversely correlated with creatinine clearance (ρ = -0.58, *P *< 0.0001) (Figures S1 and S2 of Additional data file 1).

**Table 1:** Patient characteristics and factors associated with cardiac dysfunction at intensive care unit admission

Variables	All patients	Cardiac dysfunction	No cardiac dysfunction	Odds ratio	95% CI	*P *value
	n = 198	n = 102	n = 96			

						

Male gender	130 (65.7%)	70 (68.6%)	60 (62.5%)	1.31	0.72–2.36	0.36

						

Age	67 (53–77)	73.5 (60–79)	58.5 (45–72)	1.04	1.02–1.06	< 0.0001
< 50 years	31 (15.7%)	8 (7.8%)	23 (24.0%)	0.27	0.11–0.64	0.003
50–70 years	78 (39.4%)	34 (33.3%)	44 (45.8%)	0.59	0.33–1.05	0.07
> 70 years	89 (44.9%)	60 (58.8%)	29 (30.2%)	3.30	1.83–5.94	< 0.0001

						

SAPS II at ICU admission [9]	37 (26–58)	45 (30–65)	32 (22–50)	1.03	1.01–1.04	0.0001

OSF score at ICU admission [8]	1 (0–2)	1 (0–2)	1 (0–1)	1.62	1.21–2.16	0.0001

						

Comorbidities						

Chronic cardiac failure	42 (21.2%)	28 (27.5%)	14 (14.6%)	2.22	1.08–4.53	0.29

Chronic renal failure	101 (51.0%)	64 (62.7%)	37 (34.7%)	3.01	1.65–5.48	0.0003

Creatinine clearance, mL/minute	48 (25–76)	36 (11–65)	50 (37–96)	0.98	0.98–0.99	0.0006

						

Organ failure according to OSF score						

Cardiovascular	50 (25.2%)	36 (35.3%)	14 (14.6%)	3.74	1.57–6.39	0.001
Respiratory	63 (31.8%)	38 (37.2%)	25 (26.0%)	1.66	0.90–3.07	0.10
Renal	50 (25.2%)	34 (33.3%)	16 (16.7%)	2.48	1.26–4.89	0.008
Neurological	43 (21.7%)	27 (26.5%)	16 (16.7%)	1.78	0.88–3.57	0.10
Hepatic	8 (4.0%)	4 (3.9%)	4 (3.1%)	0.93	0.22–3.82	0.91

						

Clinically documented infection	73 (36.9%)	38 (37.2)	35 (36.4%)	0.99	0.55–1.79	0.98

						

NT-proBNP per 100 ng/L	26.3 (4.7–73.0)	57.2 (14.3–157.0)	8.5 (1.9–35.6)	1.01	1.01–1.02	< 0.0001

Troponin (ng/mL)	0.10 (0.02–0.44)	0.16 (0.05–1.1)	0.06 (0.01–0.21)	1.39	1.03–1.88	0.03

ECG modifications	92 (46.5%)	75 (73.5%)	17 (17.7%)	12.90	6.51–25.59	< 0.0001

C-reactive protein (mg/L)	186 (78–332)	234 (84–370)	156 (74–223)	1.003	0.98–1.01	0.3

						

Inotropic agents	43 (21.7%)	40 (39.2%)	3 (3.1%)	20	5.92–67.51	< 0.0001
Vasopressive agents	52 (26.3%)	37 (36.3%)	15 (15.6%)	3.74	1.55–6.86	0.0013

						

Length of ICU stay, days	3 (1–8)	4 (1–10)	3 (1–6)	1.17	0.98–1.52	0.34

Length of hospital stay, days	10 (3–21)	11 (4.5–23.5)	10 (3–20)	1.01	0.99–1.01	0.39

ICU mortality	47 (23.7%)	35 (34.3%)	12 (12.5%)	3.65	1.76–7.59	0.0005

Hospital mortality	72 (36.3%)	52 (51%)	20 (20.8%)	3.95	2.1–7.4	< 0.0001

Data are presented as medians and quartiles (interquartile ranges) or as numbers (percentages). CI, confidence interval; ECG, electrocardiogram; ICU, intensive care unit; NT-proBNP, N-terminal-pro-B-type natriuretic peptide; OSF, organ system failure [8]; SAPS II, Simplified Acute Physiology Score II [9].

**Figure 1:**
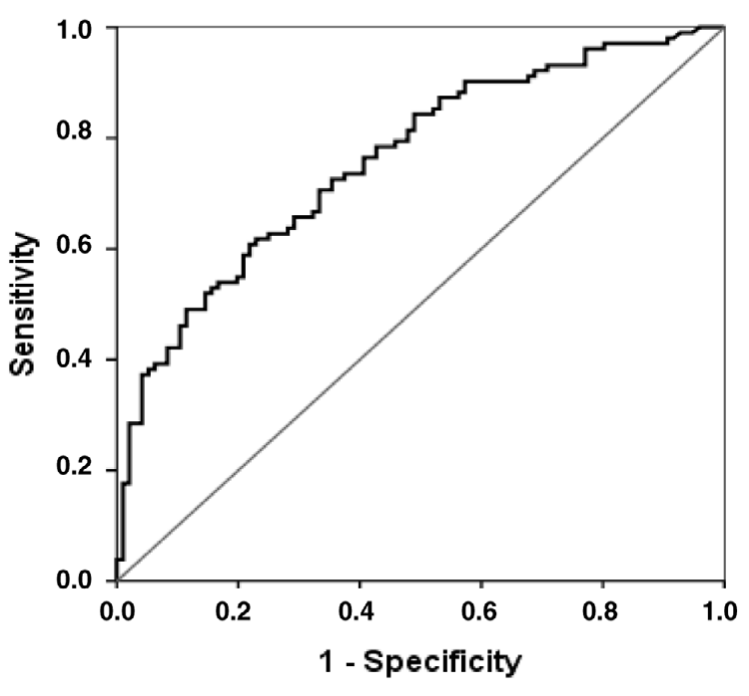
Receiver operating characteristic (ROC) curve for N-terminal-pro-B-type natriuretic peptide (NT-proBNP) in the entire cohort of critically ill patients. The ROC curve depicts the relationship between the proportion of true positives (Sensitivity) and the proportion of false positives (1 – Specificity) of different thresholds of NT-proBNP concentrations when tested to predict cardiac dysfunction. Diagonal segments are produced by ties. The area under the ROC curve was 0.76 (95% confidence interval 0.69 to 0.83).

**Figure 2:**
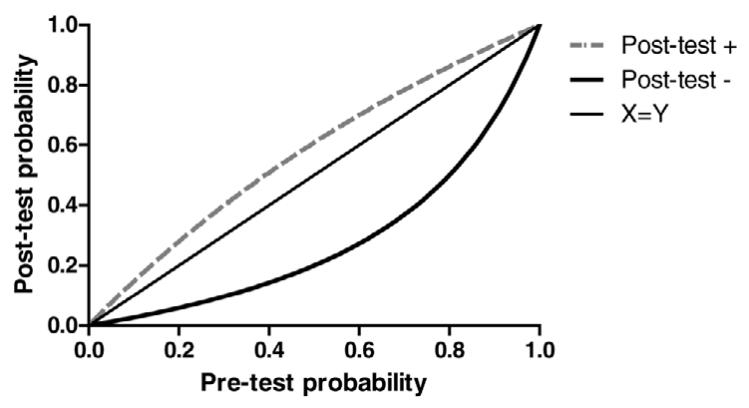
Predictive value of N-terminal-pro-B-type natriuretic peptide (NT-proBNP) for cardiac dysfunction. The cutoff of retained NT-proBNP concentrations was 500 ng/L. The estimated post-test probabilities for different pre-test probabilities are shown, assuming constancy of the positive likelihood ratio for NT-proBNP of greater than 500 ng/L (Post-test +) or of the negative likelihood ratio for NT-proBNP of less than 500 ng/L (Post-test -). Pre-test probability and post-test probability were defined, respectively, as the probabilities of cardiac dysfunction before and after a diagnostic test (NT-proBNP) result was known.

The area under the ROC curve was 0.76 (95% CI 0.69 to 0.83) (Figure 1). Performance of NT-proBNP in patients with and without acute renal failure is reported in Additional data file 1 (Figure S3a, S3b). In the overall population of patients, NT-proBNP of less than 500 ng/L predicted the absence of cardiac dysfunction with a sensitivity of 89% and a specificity of 43%. In accordance with the obtained LHs (positive LH = 1.55, negative LH = 0.25), Figure 2 depicts the ability of an NT-proBNP to modify probabilities of having a cardiac dysfunction and therefore to convert the estimated probability of the suspected diagnosis before the test result was known (pre-test probability) into a post-test probability, which takes the result into account. For example, for a pre-test probability of having a cardiac dysfunction of 20%, a negative result of the enzyme-linked immunosorbent assay test for NT-proBNP would produce a post-test probability of 5.8%. Conversely, for a pre-test probability of 70%, a negative result would yield a post-test probability of 37%.

In addition, a stepwise logistic regression model revealed that NT-proBNP (OR = 1.01 per 100 ng/L, 95% CI 1.002 to 1.02), ECG modifications (OR = 11.03, 95% CI 5.19 to 23.41), and OSF score (OR = 1.63 per point, 95% CI 1.17 to 2.41) adequately predicted cardiac dysfunction. The goodness-of-fit of the model was acceptable (Hosmer-Lemeshow: χ^2 ^= 5.27, degrees of freedom = 8, *P *= 0.728), and the area under the ROC curve of this model was 0.834 (95% CI 0.77 to 0.90). When age, creatinine clearance, troponin, or sepsis was forced into the final model, these variables did not change the model. Lastly, in our study, NT-ProBNP was not independently associated with outcome (Table S1 and Figure S4 of Additional data file 1).

## Discussion

This study, which is the largest of its kind in critically ill patients, demonstrates the relevance of NT-proBNP in the exclusion of cardiac dysfunction at ICU admission. However, our study also confirms the variability of NT-proBNP in critically ill patients and illustrates the limits to its interpretation.

### Association of NT-proBNP with age and renal function

We investigated the possible effects of age and creatinine clearance on NT-proBNP levels. The presence of renal insufficiency or advanced age was correlated with NT-proBNP concentration. This finding is consistent with previous reports [4]. We showed that, despite the effects of age and creatinine clearance on NT-proBNP levels, a single measurement of the NT-proBNP level at ICU admission might rule out cardiac dysfunction in this population, independently of age or renal function.

### Performance of NT-proBNP

Over the past decade, several studies have indicated that cardiac dysfunction is a frequent and important factor that determines the outcome of critically ill patients [13]. It has been estimated that as many as 15% to 30% of ICU admissions are complicated by some degree of myocardial injury and that as many as 40% of patients with severe sepsis have cardiac dysfunction. Therefore, measurement of NT-proBNP might be a useful tool to rule out cardiac dysfunction. However, this large cohort study confirms the large dispersion of NT-proBNP values at ICU admission. Indeed, NT-proBNP levels are higher and more variable in our study than in selected populations of patients with ischemic heart disease or in the emergency department [13,14]. This fact might explain the relatively poor positive predictive value in our study. In addition, our results confirm that careful evaluation of the pre-test probability is required to interpret the NT-proBNP measurement correctly.

### Limits

Our study has several limitations. First, the decision to evaluate a non-selected population of critically ill patients might be criticized. Indeed, the selection of patients on the basis of reason for ICU admission or of suspected cardiac dysfunction might modify the incidence of cardiac dysfunction and, therefore, the levels of NT-proBNP. However, the objective of this study was to evaluate NT-proBNP at ICU admission and not in a selected population of patients with acute respiratory failure. Another limitation is the interpretation of creatinine clearance in the ICU. Although we agree that several biases limit the interpretation of such evaluations of renal function, our results show an association between calculated creatinine clearance and NT-proBNP levels and are in accordance with those of previous studies [15,16]. Echocardiography was performed at various times during ICU stay, including at discharge in several of the patients. This design may have induced a bias and may have led to an underestimation of the accuracy of the investigational marker. Nevertheless, none of the included patients developed any significant cardiac event that may have affected the results of the echocardiography. Lastly, NT-proBNP was measured only at ICU admission. Indeed, time course of NT-proBNP may be interesting and helpful to diagnose cardiac dysfunction. However, the main objective of this study was to evaluate the diagnostic performance of the marker at ICU admission in an unselected population of critically ill patients.

## Conclusion

The present study, which examined a cohort of unselected patients admitted to the ICU, indicates that NT-proBNP is useful to exclude cardiac dysfunction in this population. However, the high variability and dispersion of NT-proBNP values necessitate careful evaluation of such biomarkers in this setting in order to interpret the results correctly. Additional studies are needed in order to evaluate time course of NT-proBNP in critically ill patients and to confirm our results.

## Key messages

• The presence of renal insufficiency or advanced age was correlated with N-terminal-pro-B-type natriuretic peptide (NT-proBNP) concentration.

• Despite this correlation, a single measurement of the NT-proBNP level at ICU admission allowed us to rule out cardiac dysfunction in the studied population, independently of age or renal function.

• Our cohort study confirms the large dispersion of NT-proBNP values at ICU admission in a non-selected population of critically ill patients.

• Careful evaluation of the pre-test probability is required to interpret the NT-proBNP measurement correctly.

## Abbreviations

BNP: B-type natriuretic peptide; CI: confidence interval; ECG: electrocardiogram; EF: ejection fraction; ICU: intensive care unit; IRB: institutional review board; LH: likelihood ratio; LV: left ventricular; NT-proBNP: N-terminal-pro-B-type natriuretic peptide; OR: odds ratio; OSF: organ system failure; ROC: receiver operating characteristic.

## Competing interests

The authors declare that they have no competing interests.

## Authors' contributions

IC participated in study concept and design, acquisition of data, analysis and interpretation of data, drafting of the manuscript, and critical revision of the manuscript for important intellectual content and had full access to all of the data in the study and takes responsibility for the integrity of the data and the accuracy of the data analysis. J-PQ participated in study concept and design, acquisition of data, analysis and interpretation of data, drafting of the manuscript, and critical revision of the manuscript for important intellectual content. BB participated in study concept and design, acquisition of data, and critical revision of the manuscript for important intellectual content. J-MD, M Degrés, and PG participated in acquisition of data. M Darmon participated in analysis and interpretation of data, drafting of the manuscript, and critical revision of the manuscript for important intellectual content and performed statistical analysis. BS participated in critical revision of the manuscript for important intellectual content. All authors read and approved the final manuscript.

## Supplementary Material

Additional file 1 **Table S1: **Factor independently associated with hospital mortality when introduced in a logistic regression model; **Figure S1: **Relationship between N-terminal-pro-B-type natriuretic peptide (NT-proBNP) level and creatinine clearance; **Figure S2: **Relationship between N-terminal-pro-B-type natriuretic peptide (NT-proBNP) and patient age; **Figure S3a: **Accuracy of N-terminal-pro-B-type natriuretic peptide (NT-proBNP) measurement for diagnosis of cardiac dysfunction in patients without renal failure; **Figure S3b: **Accuracy of N-terminal-pro-B-type natriuretic peptide (NT-proBNP) measurement for diagnosis of cardiac dysfunction in patients with acute renal failure; **Figure S4: **Accuracy of N-terminal-pro-B-type natriuretic peptide (NT-proBNP) measurement for the prediction of hospital death in the overall population. Click here for file

## References

[B1] Brueckmann M, Huhle G, Lang S, Haase KK, Bertsch T, Weiss C, Kaden JJ, Putensen C, Borggrefe M, Hoffmann U (2005). Prognostic value of plasma N-terminal pro-brain natriuretic peptide in patients with severe sepsis. Circulation.

[B2] Januzzi JL, Chen-Tournoux AA, Moe G (2008). Amino-terminal pro-B-type natriuretic peptide testing for the diagnosis or exclusion of heart failure in patients with acute symptoms. Am J Cardiol.

[B3] McLean AS, Huang SJ, Hyams S, Poh G, Nalos M, Pandit R, Balik M, Tang B, Seppelt I (2007). Prognostic values of B-type natriuretic peptide in severe sepsis and septic shock. Crit Care Med.

[B4] McLean AS, Huang SJ, Nalos M, Tang B, Stewart DE (2003). The confounding effects of age, gender, serum creatinine, and electrolyte concentrations on plasma B-type natriuretic peptide concentrations in critically ill patients. Crit Care Med.

[B5] Mueller C (2007). The use of B-type natriuretic peptides in the intensive care unit. Crit Care Med.

[B6] Mueller C, Scholer A, Laule-Kilian K, Martina B, Schindler C, Buser P, Pfisterer M, Perruchoud AP (2004). Use of B-type natriuretic peptide in the evaluation and management of acute dyspnea. N Engl J Med.

[B7] Green SM, Martinez-Rumayor A, Gregory SA, Baggish AL, O'Donoghue ML, Green JA, Lewandrowski KB, Januzzi JL (2008). Clinical uncertainty, diagnostic accuracy, and outcomes in emergency department patients presenting with dyspnea. Arch Intern Med.

[B8] Knaus WA, Draper EA, Wagner DP, Zimmerman JE (1985). Prognosis in acute organ-system failure. Ann Surg.

[B9] Le Gall JR, Lemeshow S, Saulnier F (1993). A new Simplified Acute Physiology Score (SAPSII) based on a European/North American multicenter study. JAMA.

[B10] Bone RC, Sibbald WJ, Sprung CL (1992). The ACCP-SCCM consensus conference on sepsis and organ failure. Chest.

[B11] Bewick V, Cheek L, Ball J (2004). Statistics review 13: receiver operating characteristics curves. Crit Care.

[B12] Deeks J (2001). Systematic reviews in health care: systematic reviews in evaluation of diagnosis and screening tests. BMJ.

[B13] Fuat A, Murphy JJ, Hungin AP, Curry J, Mehrzad AA, Hetherington A, Johnston JI, Smellie WS, Duffy V, Cawley P (2006). The diagnostic accuracy and utility of a B-type natriuretic peptide test in a community population of patients with suspected heart failure. Br J Gen Pract.

[B14] Battaglia M, Pewsner D, Juni P, Egger M, Bucher HC, Bachmann LM (2006). Accuracy of B-type natriuretic peptide tests to exclude congestive heart failure: systematic review of test accuracy studies. Arch Intern Med.

[B15] Karmpaliotis D, Kirtane AJ, Ruisi CP, Polonsky T, Malhotra A, Talmor D, Kosmidou I, Jarolim P, de Lemos JA, Sabatine MS, Gibson CM, Morrow D (2007). Diagnostic and prognostic utility of brain natriuretic Peptide in subjects admitted to the ICU with hypoxic respiratory failure due to noncardiogenic and cardiogenic pulmonary edema. Chest.

[B16] Rudiger A, Gasser S, Fischler M, Hornemann T, von Eckardstein A, Maggiorini M (2006). Comparable increase of B-type natriuretic peptide and amino-terminal pro-B-type natriuretic peptide levels in patients with severe sepsis, septic shock, and acute heart failure. Crit Care Med.

