# Supplementary material for: Performance of N-terminal-pro-B-type natriuretic peptide in critically ill patients: a prospective observational cohort study
Source: Crit Care. 2008 Nov 6;12(6):R137. doi: 10.1186/cc7110 (PMC2646347; doi:10.1186/cc7110)
Supplement: Additional file 1 — Table S1: Factor independently associated with hospital mortality when introduced in a logistic regression model; Figure S1: Relationship between N-terminal-pro-B-type natriuretic peptide (NT-proBNP) level and creatinine clearance; Figure S2: Relationship between N-terminal-pro-B-type natriuretic peptide (NT-proBNP) and patient age; Figure S3a: Accuracy of N-terminal-pro-B-type natriuretic peptide (NT-proBNP) measurement for diagnosis of cardiac dysfunction in patients without renal failure; Figure S3b: Accuracy of N-terminal-pro-B-type natriuretic peptide (NT-proBNP) measurement for diagnosis of cardiac dysfunction in patients with acute renal failure; Figure S4: Accuracy of N-terminal-pro-B-type natriuretic peptide (NT-proBNP) measurement for the prediction of hospital death in the overall population. [file cc7110-S1.doc]

Performance of N-terminal-pro-B-type natriuretic peptide in critically ill patients: a prospective observational cohort study

**Isaline Coquet et al.**

**Online data supplement**

**Table S1.** Factors associated with hospital mortality. Logistic regression analysis was performed to identify variables that were associated significantly with hospital mortality, as measured by the estimated odds ratio (OR) with 95% confidence interval (95% CI). Variables yielding *p* values <0.20 in the bivariate analyses were entered into a forward stepwise logistic regression model in which hospital mortality was the variable of interest. The covariates were entered into the model with critical removal *p* values of 0.1. Colinearity and interactions were tested. The Hosmer-Lemeshow test was used to check goodness-of-fit of the logistic regression. When NT-proBNP was forced into the final model, it did not change the final model

|  | **Odds ratio** | **95% CI** | ***p* value** |
| --- | --- | --- | --- |
|  |  |  |  |
| **Age** | **1.02** | **0.99-1.05** | **0.07** |
| **Sepsis at ICU admission** | **0.28** | **0.07-1.11** | **0.07** |
| **OSF score (per point)** | **2.84** | **1.88-4.29** | **0.001** |

**95% CI, 95% confidence interval; ICU, intensive care unit; OSF, Organ-System Sepsis Failure.**

Area under the receiver operating characteristic curve = 0.73 (95% CI 0.63−0.86);

Hosmer-Lemeshow goodness-of-fit (χ² = 7.42; df = 8; *p* = 0.498).

**Fig. S1.** Relationship between NT-proBNP level and creatinine clearance. NT-proBNP levels at ICU admission in patients with (dark grey) and without (light grey) cardiac dysfunction according to creatinine clearance. Compared to patients with cardiac dysfunction, patients without cardiac dysfunction had lower NT-proBNP levels (ANOVA test; *p* < 0.0001)

**Fig. S2.** Relationship between NT-proBNP and patient age. NT-proBNP levels at ICU admission in patients with (dark grey) and without (light grey) cardiac dysfunction according to their age. Compared to patients with cardiac dysfunction, patients without cardiac dysfunction had lower NT-proBNP levels (ANOVA test; *p* < 0.0001)

**Fig. S3a.** Accuracy of NT-proBNP measurement for diagnosis of cardiac dysfunction in patients without renal failure. The receiver operating characteristic (ROC) curve for patients without renal failure (defined as a creatinine clearance <60 ml/min) depicts the relationship between the proportion of true positives (Sensitivity) and the proportion of false positives (1 - Specificity) of different thresholds of NT-proBNP concentrations when tested to predict cardiac dysfunction. The area under the ROC curve was 0.76 (95% CI 0.69−0.83)

**Fig. S3b.** Accuracy of NT-proBNP measurement for diagnosis of cardiac dysfunction in patients with acute renal failure. The receiver operating characteristic (ROC) curve for patients with renal failure (defined as a creatinine clearance <60 ml/min) depicts the relationship between the proportion of true positives (Sensitivity) and the proportion of false positives (1 - Specificity) of different thresholds of NT-proBNP concentrations when tested to predict cardiac dysfunction. The area under the ROC curve was 0.74 (95% CI 0.64−0.84)

**Fig. S4.** Accuracy of NT-proBNP measurement for the prediction of hospital death in the overall population. The receiver operating characteristic (ROC) curve for the overall population depicts the relationship between the proportion of true positives (Sensitivity) and the proportion of false positives (1 - Specificity) of different thresholds of NT-proBNP concentrations when tested to predict hospital mortality. The area under the ROC curve was 0.64 (95% CI 0.55−0.73).

**Figure S1.**

**
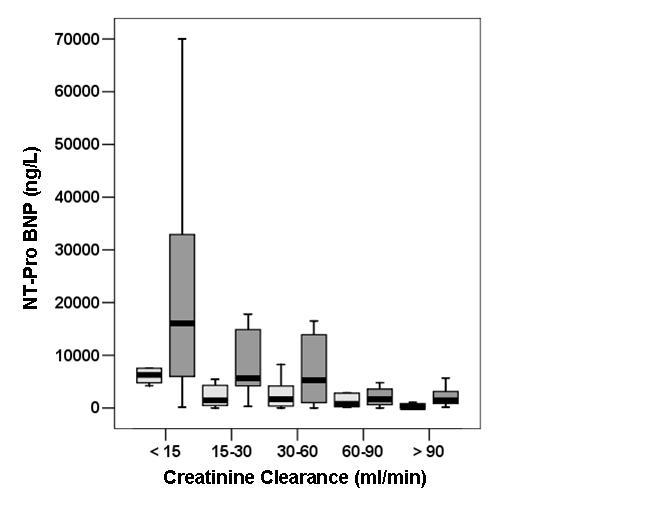
**

**Figure S2.**

**
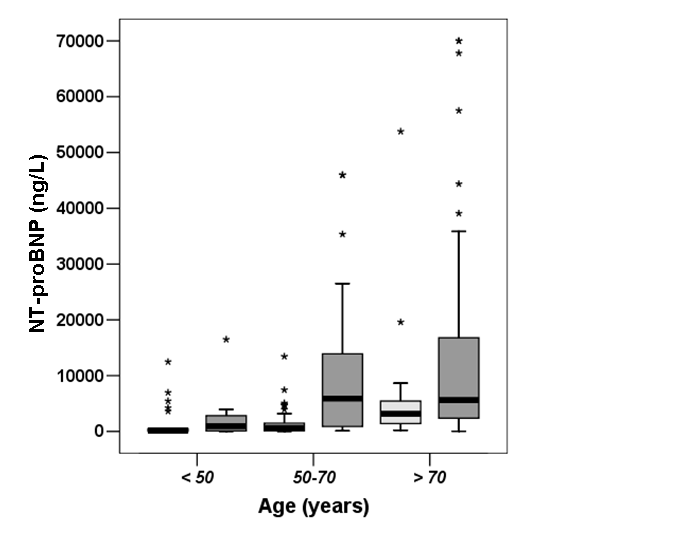
**

**Figure S3a.**

**Figure S3b.**

**Figure S4.**

**Figure S4.**
